# microRNA Fine-Tuning of the Germinal Center Response

**DOI:** 10.3389/fimmu.2021.660450

**Published:** 2021-04-19

**Authors:** Teresa Fuertes, Irene Salgado, Virginia G. de Yébenes

**Affiliations:** ^1^ B Lymphocyte Biology Lab, Centro Nacional de Investigaciones Cardiovasculares, Madrid, Spain; ^2^ Department of Immunology, Ophthalmology and ENT, Universidad Complutense de Madrid School of Medicine, Madrid, Spain; ^3^ Inmunología Linfocitaria Lab, Hospital 12 de Octubre Health Research Institute (imas12), Madrid, Spain

**Keywords:** microRNA, germinal center, antibodies, autoimmunity, neoplasia

## Abstract

Germinal centers (GCs) are complex multicellular structures in which antigen-specific B cells undergo the molecular remodeling that enables the generation of high-affinity antibodies and the differentiation programs that lead to the generation of plasma–antibody-secreting cells and memory B cells. These reactions are tightly controlled by a variety of mechanisms, including the post-transcriptional control of gene expression by microRNAs (miRNAs). Through the development of animal models with B cell-specific modified miRNA expression, we have contributed to the understanding of the role of miRNAs in the regulation of GC responses and in B cell neoplasia. Here, we review recent advances in the understanding of the role of miRNAs in the regulation of B cell and T follicular helper physiology during the GC response and in the diseases associated to GC response dysregulation.

## Introduction

The germinal center (GC) response is a key B lymphocyte maturation and differentiation program essential for the generation of competent protective immunity. The GC response is initiated in mature B lymphocytes after antigen encounter and leads to the generation of memory B cells and plasma antibody-secreting cells that produce antibodies with high antigen affinity and with different immunoglobulin (Ig) isotypes, conferring the Ig molecule with the ability to orchestrate different immune effector responses (1, 2). At the molecular level, these reactions are initiated by the activity of activation induced deaminase (AID), an enzyme that deaminates cytosines in the Ig genes, triggering somatic hypermutation (SHM) and class switch recombination (CSR), processes respectively responsible for the changes in affinity and isotype in the Ig genes. At the cellular level, initiation of the GC reaction requires the cognate interaction of antigen-activated B-lymphocytes with a specialized subset of GC T CD4 cells, the follicular T helper (Tfh) cells. Tfh-GC B cell interactions are dependent on a number of molecule interactions that signal for full B and Tfh cell differentiation together with cellular localization in follicles. These interactions include T-cell receptor recognition of B cell peptide-MHC complexes as well as CD40 and ICOS ligand co-receptor interactions (3). Developing Tfh and B GC cells are influenced by changing cytokine, chemokine and cellular environments through the induction of specific transcriptional programs (4). Gene transcription in Tfh and B cells is regulated by key GC transcription factors such as BCL6, as well as by RNA-binding proteins and microRNAs (miRNAs) (3, 5). miRNAs are small non-coding RNA molecules that drive post-transcriptional negative regulation of gene expression by promoting the degradation or translational blockade of partially complementary target mRNAs. Mature miRNAs are 21-24-nucleotide RNA molecules processed from longer RNA precursors in two consecutive cleavage steps mediated by the RNase III enzymes Drosha and Dicer (6). Ablation of miRNAs in miRNA-processing-enzyme deletion knockout models has demonstrated that miRNAs play essential roles in diverse developmental, cellular, and physiological processes (7, 8). miRNAs fine-tune cellular gene expression networks and have emerged as essential regulators of GC differentiation responses.

## miRNAs in Physiological GC Regulation

**Figure 1:**
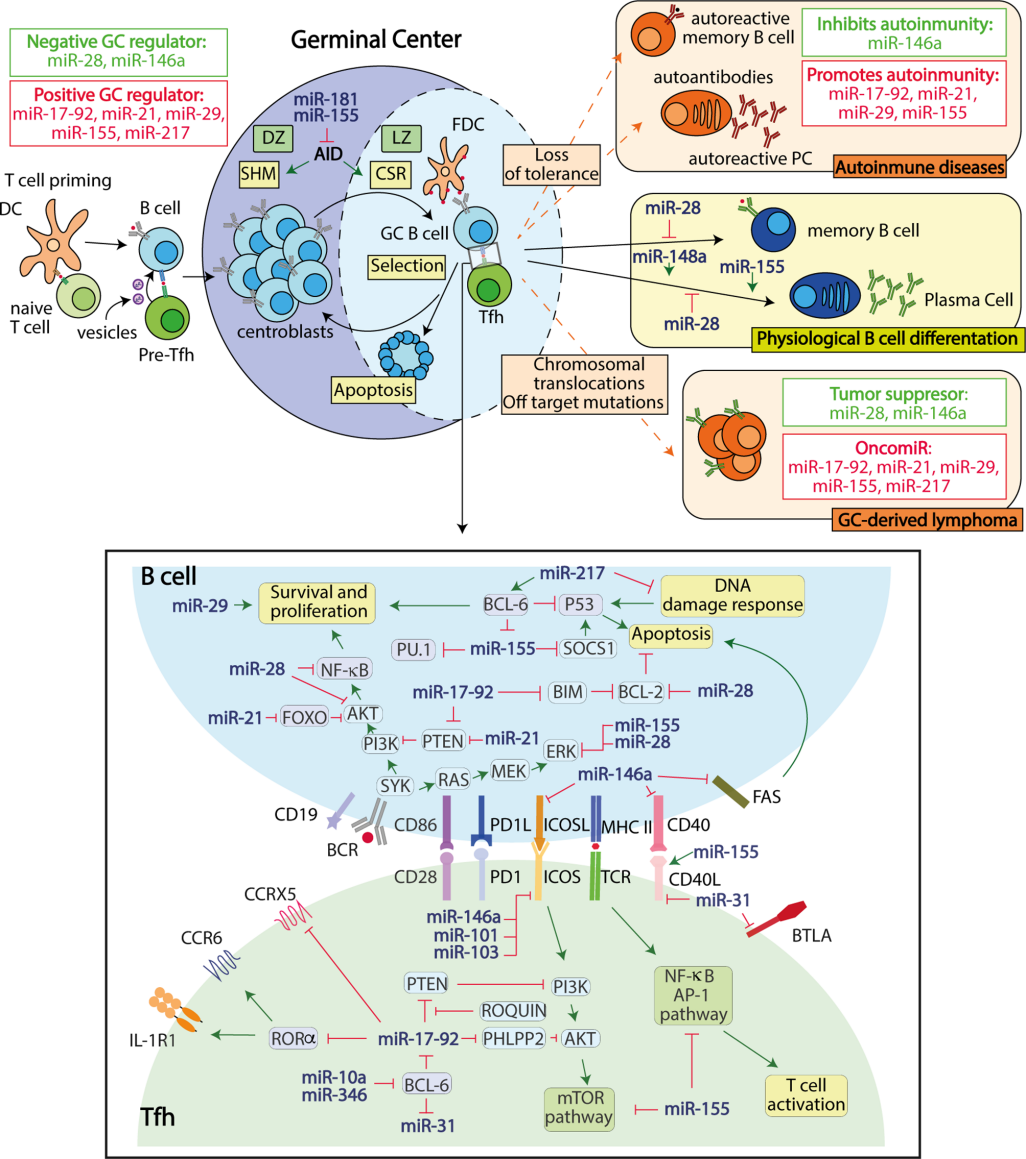
miRNAs regulate gene expression in B and Tfh germinal center cells. Regulated miRNA expression is required to regulate B-Tfh cell interactions and ensure proper GC responses. GC-derived dysfunctions caused by miRNA alterations can lead to the development of autoimmunity and/or B cell neoplasia through the disruption of post-transcriptional control mechanisms required for the maintenance of GC homeostasis.

Studies of global miRNA depletion in GC B and T cell-specific models showed that miRNAs are essential for proper GC formation (9, 10). Dicer-mediated miRNA depletion after AID expression in early activated GC B cells impaired the production of high-affinity class-switched antibodies and the generation of memory B and long-lived plasma cells after T cell-dependent immunization due to defects in B cell proliferation and survival (9). Likewise, DGCR8-Drosha complex-mediated miRNA depletion in CD4 T cells showed that CD4 T cell-expressed miRNAs are essential for the differentiation of Tfh cells and the induction of GC B cells during T cell-dependent immunizations (10). Interestingly, miRNAs are not only required to regulate Tfh and GC B cell function in a cell intrinsic manner, but are also important contact-independent mediators of T-B cellular communication (**Figure 1**). This communication occurs through the transfer to B cells of a restricted set of T cell-derived miRNAs in extracellular vesicles and modulates the efficiency of GC generation and antibody secretion in response to immunization (11).

### miRNAs in the Regulation of B Cells in the GC

The most extensively studied GC B cell miRNA is miR-155, whose expression is upregulated after mature B cell activation and in GC B cells (12–15). Infection of miR-155-deficient mice with pathogenic bacteria showed that miR-155 expression is required to control pathogen-induced disease (16). Characterization of the response to T cell-dependent immunizations in miR-155^-/-^ loss-of-function and miR-155^KI^ gain-of-function mouse models revealed that miR-155 expression is required for efficient adaptive immune responses, including the generation of GC B cells and the secretion of antigen-specific antibodies (12, 16). miR-155 is a positive regulator of the GC response, and deficiency in miR-155 expression leads to reduced cytokine production, IgG1 secretion, impaired affinity maturation, and plasmablast B cell generation in a B cell autonomous manner (12, 17, 18). miR-155 controls affinity-based selection, at least in part, by protecting light zone (LZ) GC c-MYC^+^ B cells from apoptosis (19).

Transcriptome studies showed that miR-155 regulates the expression of numerous mRNAs in B cells (17, 18), although the functional consequences of miR-155-dependent mRNA regulation in GC B cells has been characterized for only a few miR-155 targets. The transcription factor PU.1 is a direct miR-155 target implicated in miR-155 mediated effects on CSR (17). PU.1 is encoded by *Sfpi1*, and the consequences of disrupting miR-155–*Sfpi1* mRNA interaction *in vivo* were determined by generating knock-in mice with a mutation in the miR-155 recognition site in the *Sfpi1* mRNA 3’UTR. miR-155-mediated PU.1 post-transcriptional regulation was shown to be required for efficient terminal plasma B cell differentiation and antigen-specific immunoglobulin (Ig) secretion through the downregulation of *Pax5* expression and genes involved in adhesion and B-T cell interactions (20).

The other well characterized miR-155 target in GC B cells is activation-induced deaminase (AID), the enzyme responsible for the molecular remodeling of Igs in the GC. Knock-in mice with a disruption of the miR-155 recognition site in the *Aicda* mRNA 3’UTR demonstrated that miR-155 expression in GC B cells is needed to limit AID expression, allow proper affinity maturation, and restrict oncogenic AID-mediated MYC-IgH chromosomal translocations (21, 22). GC tolerance of DNA damage is multilayered and temporally regulated (23), and miR-155 expression is in turn limited by the expression of BCL6 (24, 25), an important transcriptional regulator and proto-oncogene that inhibits the DNA damage response in GC B cells (26). In addition, miR-155 negatively regulates the expression of *Socs1*, a P53 activator important for the DNA damage response (27). miR-155 thus plays a dual role in modulating the accumulation of DNA double-strand breaks (DSB) associated with the GC reaction, regulating P53 activity by controlling the expression levels of *Aicda* and *Socs1*.

AID expression is directly regulated in B cells by yet other miRNAs in B cells. miR-361 is another BCL6-downregulated miRNA that targets *Aicda*, presumably in light-zone GC B cells (25). miR-181b, which is highly expressed in mature resting B cells and whose expression diminishes upon B cell activation, targets *Aicda* directly through the binding of several partly complementary sequences found in its mRNA 3’UTR (28). Thus, AID levels are controlled by different miRNAs at different stages of B cell activation.

Another miRNA that positively regulates the GC response upon its induction during B cell activation and in GC B cells is miR-217. Using gain- and loss-of-function mouse models, we showed that miR-217 promotes the generation of GC B cells and increases the generation of class-switched antibodies and the frequency of somatic hypermutation in B cells. We found that miR-217 regulates a DNA damage response and repair gene network that stabilizes BCL6 expression in GC B cells (29). Thus, miR-217 downregulates a network of genes that sense and repair genotoxic events on DNA, which in turn can increase GC B cell tolerance to DNA damage in the context of AID activity, very much like BCL6. Notably, we found that miR-217 protects BCL6 from previously described genotoxic stress-induced degradation (23), suggesting that both molecules form part of the same network that renders GC cells permissive to genomic instability and prone to malignant transformation.

Positive regulation of terminal post-GC plasma B cell differentiation has been suggested to be regulated by other miRNAs. A likely candidate is miR-148a, the most abundant miRNA in human and murine plasma cells, which has been shown to promote plasma cell differentiation and survival *in vitro.* Importantly, miR-148a expression was shown to downregulate the expression of the GC transcription factors *Mitf* and *Bach2*, which block premature plasma cell maturation and favor cell death (30). Definition of the role of miR-148a as a regulator of GC-dependent plasma cell differentiation *in vivo* would require the development of gain- or loss-of-function miR-148a B cell-specific mouse models.

GC miRNAs can also act as regulators that restrict the GC response, the best-characterized negative regulators of GC responses being miR-28 and miR-146a. miR-28 is a GC-specific miRNA (14, 15) whose expression is lost in numerous mature B-cell neoplasms (31–33). By combining gain- and loss-of-function approaches, we showed that miR-28 negatively regulates CSR and immunization-triggered GC and post-GC plasma and memory B cell generation. Combined transcriptome and proteome analysis upon inducible re-expression of miR-28 in B cells revealed that miR-28 expression induces the coordinated downregulation of the key BCR signaling gene network regulating B-cell proliferation and cell death (33), thus supporting the notion that miR-28 limits the strength of BCR signaling and regulates proliferation and survival of GC B cells.

miR-146a is expressed in B cells upon stimulation and within GC B cells (15), and loss of miR-146a causes a B cell-intrinsic increase in the GC response to immunization (34), spontaneous GC generation in aged mice, and increased production of anti-double–stranded DNA (dsDNA) auto-antibodies (35). miR-146a was shown to limit B cell GC functional responses by downregulating B cell expression of signaling pathway components involved in GC B Tfh cellular interactions, such as ICOSL (34) and CD40 (35).

Other miRNAs have also been suggested to negatively regulate terminal post-GC plasma and memory B cell differentiation. miR-125b, a miRNA highly expressed in dividing centroblasts in GC B cells (36), has been shown to inhibit plasma cell generation and antibody secretion *in vitro* (37, 38). Importantly, direct mRNA targeting by miR-125b was shown to downregulate the expression of BLIMP-1 and IRF-4 transcription factors, which are essential for plasma cell differentiation (36–39). *Prdm1*, the gene encoding BLIMP-1, is a direct target of other highly expressed GC B cell miRNAs, including miR-9, miR-30a, and let-7 family miRNAs (40–43). Interestingly, the expression of miR-30a and miR-125b is regulated epigenetically in B cells and can be modulated using histone deacetylase inhibitors to inhibit BLIMP-1 expression in the context of antibody responses and GC-derived diseases (44–46). Memory B cell generation is associated to changes in chromatin accessibility and miRNA expression, and miR-181 has been recently identified as a major gene expression regulator during memory B cell differentiation (47).

Overall, these studies have identified a set of miRNAs that are required to promote or limit the GC reaction through post-transcriptional gene expression regulation in B cells (**Figure 1** and **Table 1**).

**Table 1:** Identified roles of miRNAs in the regulation of physiological GC responses and in GC-derived dysfunctions.

GC regulation miRNA	Role in GC physiology	Role in B cell neoplasia	Role in autoimmunity	Molecular mechanisms and targets
miR-17-92 polycistron (miR-17, miR-18a, miR-19a, miR-20a, miR-19b, and miR-92a)	** Positive GC regulator**. Promotes GC responses, Tfh and GC B cell generation (10, 48, 49).	**OncomiR.** Promotes B cell GC-derived lymphoma (50)	**Promotes autoimmunity.** miR-17-92 expression in lymphocytes promotes spontaneous accumulation of Tfh and GC B cells, IgG anti-dsDNA antibodies and fatal immunopathology (48, 51).	Promotes proliferation and survival in lymphocytes by inhibiting the expression of *Pten* and *Bim* (51). Regulates differentiation and enhances ICOS-PI3K signaling by downregulating *Pten* and *Phlpp2* phosphatase gene expression in Tfh cells (10, 48, 49).
miR-155	** Positive GC regulator** Promotes GC responses and Tfh and GC B cell generation (12, 16, 52)	**OncomiR** Induces preB and mature B cell lymphomas (53–56)	**Promotes autoimmunity** miR-155 expression promotes autoimmunity in autoimmune mouse models of collagen-induced arthritis (57), systemic lupus erythematosus Fas^lpr^ (58, 59), and age-dependent miR-146a deficiency (52).	Regulates the GC reaction *via* B cell-intrinsic (12, 17, 18) and T cell-intrinsic mechanisms (52). Prevents LZ GC c-MYC^+^ B cell apoptosis by downregulating *Jarid2* (19). Targets *Sfpi1* (17, 60) and *Aicda* (21, 22, 44) mRNAs and prevents P53 (27) and ERK activation through the inhibition of SHIP-1 (58) in B cells. Promotes *Prmd1*/BLIMP-1 expression and plasma cell differentation through PU.1-*Pax5* downregulation in B cells (20, 60). Regulates Tfh development and autoimmunity by modulating NF-κB, AP-1, and mTOR pathways (52) and promotes Tfh cellular proliferation and CD40L expression by repressing *Peli1* (61). Targets *S1pr1* in B cells from Fas^lpr^ lupus-like mice, and its expression is decreased in SLE patients (57, 59). Inhibits *Pu.1* in rheumatoid arthritis B cells (60). Promotes age-dependent inflammation associated to accumulation of Tfh, GC B cells and the generation of autoantibodies in miR146a deficient mice (62).
miR-217	** Positive GC regulator** Promotes GC B cell generation and GC responses (29).	**OncomiR** Overexpression in B cells leads to clonal GC-derived lymphomas (29).	NA	Downregulates DNA damage and repair response through *Nbs1*, *Xrcc2*, *Lig4*, and *Pds5b* gene expression downregulation and BCL6 stabilization (29).
miR-29	** Positive GC regulator** Promotes GC B cell generation after T-cell dependent immunization (63)	**OncomiR** Overexpression in B cells leads to B-cell chronic lymphocytic leukemia (B-CLL) development (64)	**Promotes autoimmunity** Promotes autoimmunity in collagen-induced arthritis (63)	Promotes B-cell proliferation (63, 64). Downregulates the expression of *Ddk6*, *Dnmt3a*, and the P53-responsive and tumor suppressor gene *Pxdn* (64).
miR-21	** Positive GC regulator** Promotes GC responses, Tfh and GC B cell generation (Schell SL J Immunol 2019, 202 (1 Supplement) 121.12; (Abstr) (65). Expression inhibited by BLIMP-1 during plasma cell differentiation (66).	**oncomiR** Induces B lymphomas dependent on continuous miR-21 expression (67).	**Promotes autoimmunity** miR-21 inhibition ameliorates disease in a lupus model (68)	Promotes B cell activation and proliferation. Activates the PI3K–AKT–mTOR pathway. Inhibits expression of *Pten*, *Pdcd4* (69), *Foxo* (70), *Fas* (65), and *Pdcd4* (68).
miR-28	** Negative GC regulator** Impairs CSR and memory B and plasma cell differentiation (33).	**Tumor suppressor** Efficiently inhibits tumor growth after intratumor or systemic administration of miR-28 synthetic mimics in various DLBCL and BL xenograft models and in a primary mouse BL (33).	NA	Inhibits BCR signaling and impairs B-cell proliferation and survival. Inhibits *MAD2L1, BAG1, RAP1B, p-AKT, p-ERK, NFKB2, IKKB* and *BCL2* gene expression (32, 33).
miR-146a	** Negative GC regulator** Limits Tfh and GC B cell generation and GC responses in T and B cells (34, 35, 52)	**Tumor suppressor** miR-146a knockout mice spontaneously develop B cell lymphomas and myeloid malignancies (71, 72). miR-146b and miR-146a knockout mice develop histologically distinct B cell lymphomas (73). miR-146a deficiency accelerates c-MYC-induced B cell lymphoma development (74).	**Inhibits autoimmunity** Loss of miR-146a in immune cells promotes autoimmunity (72) (52). Loss of miR-146a causes a B cell-intrinsic increase in anti- dsDNA auto-antibody production and spontaneous GC reactions (35).	Immunosuppressive roles in innate and adaptive immunity (72, 75). Downregulates the expression of signaling pathway components involved in GC B-Tfh cellular interactions, such as ICOSL-ICOS (34) and CD40-CD40L (35). Limits Tfh numbers by downregulating *Stat1* expression (75) and counterregulates miR-155 targets in Tfh cells, which is relevant to inhibit the generation of autoantibodies associated to age-dependent inflammation (52). Dysregulated overexpression promotes a lymphoproliferative syndrome and GC B cell expansion *via Fas* expression downregulation in GC B cells (76).

NA, not analyzed.

### miRNAs in the Regulation of Follicular Helper T Cells

The induction of the GC reaction is critically dependent on the colocalization of B cells with Tfh cells and interaction between the two. This GC B-Tfh cell interaction and the resulting intracellular signaling are also controlled by miRNAs expressed in Tfh cells. Remarkably, miR-146a downregulates the inducible costimulatory *Icos* expression in Tfh cells (34), and thus the expression of the two interacting molecules (ICOS and ICOSL) of this costimulatory pathway are negatively controlled in both cell subsets by the same miRNA. ICOS directly controls the migration of CD4^+^ T cells from the T cell-B cell border into the B cell follicles of peripheral lymphoid organs (77). Importantly, ICOS signaling in T cells was shown to be important for miR-146a mediated Tfh and GC regulation (34). *Icos* expression is also negatively regulated by two other miRNAs whose expression is downregulated during Tfh differentiation, miR-101 and miR-103 (78, 79). Thus, ICOS co-stimulatory receptor expression is redundantly regulated by miRNAs in Tfh cells presumably to limit or end the GC reaction.

GC B-Tfh derived ICOS signaling is mediated by the PI3K/AKT pathway (80) and inhibited by PTEN phosphatase activity in Tfh cells (81). This key signaling pathway for Tfh activation and differentiation (3) is additionally regulated at different levels in Tfh cells by miRNAs from the miR-17-92 cluster. miR-17-92 cluster expression is induced early in T cell activation (48) and is repressed by BCL6, the critical transcriptional factor that regulates Tfh differentiation (82). T cell-specific miR-17-92 gain- and loss-of function mouse models showed that the microRNAs of the cluster are critical promoters of Tfh and GC B cell differentiation and antigen-specific antibody generation during both T-cell dependent immune responses and chronic viral infection (10, 48, 49). miR-17-92 cluster miRNAs regulate the ICOS-PI3K signaling pathway in Tfh cells through the simultaneous targeting of different pathway inhibitory components. miR-17-92 inhibits PTEN phosphatase expression upstream of AKT (10, 48, 51) as well as the downstream AKT phosphatase PHLPP2 (48). This pathway is additionally regulated in Tfh cells by Roquin, an RNA-binding protein that recognizes specific stem-loop structures in the 3’UTRs of target mRNAs and which interferes with miR-17-92 binding to an overlapping binding site in the *Pten* mRNA 3’UTR (83). Important miR-17-92 targets mediating other aspects of the Tfh differentiation program include the transcription factor RORα; responsible for the induction of IL-1R1 and CCR6 expression in Tfh cells (10), and CXCR5, a hallmark Tfh molecule that influences Tfh cell localization to follicles in which the ligand CXCL13 is expressed (82).

BCL6 represses the expression of a significant fraction of the miRNAs expressed in mouse CD4^+^ T cells (82); however, the functional contribution of this repression to the Tfh cell transcriptional program has been characterized for few miRNAs outside the miR-17-92 cluster. BCL6 represses miR-31 expression in human Tfh cells through direct binding to its promoter (84). miR-31 inhibits the expression of CD40L, SAP, and BTLA, which are crucial for Tfh cell helper activity and cross-talk with B cells (85–87). Accordingly, Tfh cells forced to express miR-31 display decreased B-helper activity (84). Although BCL6 controls Tfh activity in humans and mice, the role of miR-31 is restricted to human Tfh cell differentiation, reflecting a species specificity on the action of some miRNAs.

*Bcl6* gene expression is also regulated by miRNAs in CD4^+^ T cells, and this regulation influences the generation of Tfh cells from T cell precursors. miR-10a, a miRNA highly expressed in mouse regulatory T cells (T_reg_), has been proposed to attenuate the conversion of inducible T_regs_ to Tfh cells through *Bcl6* repression in mice (88). miR-346 has been suggested to repress *BCL6* gene expression in human Tfh cells (62).

Another key miRNA regulator of both CG B and Tfh cells is the positive GC regulator miR-155. Immunization of T cell-specific miR-155-deficient *Cd4*-Cre miR155^fl/fl^ mice revealed impaired in GC B and Tfh cell generation and antigen-specific antibody production (52), revealing that miR-155 expression regulates Tfh development during immunization responses through T cell intrinsic mechanisms. The same study showed that miR-155 regulates different Tfh-cell targets important for Tfh development and autoimmunity in the NF-κB, AP-1 and mTOR pathways. Interestingly, miR-155 promotes Tfh cell accumulation during chronic, low-grade inflammation by counteracting the effect of miR-146a in Tfh cells (52). A later study showed that miR-155 promotes Tfh cell proliferation and CD40L expression by repressing expression of *Peli1*, a ubiquitin ligase that promotes the degradation of the NF-κB family transcription factor c-REL (61). These data suggest that miR-155 contributes to increased Tfh-mediated GC B activation through increased CD40L–CD40 interaction, which is known to be a limiting step in B cell clonal expansion, GC formation, isotype switching, affinity maturation, and the generation of long-lived plasma cells (89, 90).

Thus, miRNAs regulate Tfh cellular differentiation and interaction with B cells in the GC at multiple levels and through multilayer regulatory molecular circuits (**Figure 1**).

## miRNAs in GC-Derived B Cell Neoplasia and Autoimmune Diseases

Defects in GC regulation lead to immune diseases such as autoimmunity and mature B-cell neoplasia. These diseases are ultimately caused by the dysregulation of two distinct GC checkpoints; a breakdown of immune tolerance in autoimmunity and a surpassing of the DNA damage-tolerance threshold associated with Ig remodeling during CSR and SHM in B cell neoplasia. However, both diseases share a contribution from some of the mechanisms that promote GC dysfunction, including lymphoproliferative aberrant GC persistence, abnormal cellular components, and abnormal cellular signaling (91–93).

Recent studies addressing the contribution of miRNAs to these two GC-derived diseases revealed that dysregulated miRNA expression in GC B or Tfh cells can trigger B cell neoplasia or autoimmunity (**Figure 1**). Interestingly, several miRNAs that positively regulate the GC response also promote autoimmunity and B cell neoplasia (**Table 1**). For instance, miR-155, which promotes GC responses through T and B cell intrinsic mechanisms (12, 16, 52), also promotes autoimmune diseases characterized by switched auto-antibodies (52, 57–60, 94) and B cell neoplasia (53–56), likely through multilayer mechanisms that can lead to aberrant GC persistence due to increased proliferation, reduced cell death and altered cellular signaling of Tfh and GC B cells (**Table 1**). Similarly, the miR-17-92 polycistron, which promotes GC responses by enhancing Tfh and B lymphocyte proliferation and survival by inhibiting the expression of *Pten* and *Bim* (10, 48, 49, 51), when overexpressed in different mouse models promotes the generation of spontaneous GCs, IgG anti-double–stranded DNA (dsDNA) autoantibodies linked to fatal immunopathology (48, 51), and B cell GC-derived lymphoma (50). Accordingly, miR-155 and miR-17-92 are upregulated in mature B cell neoplasia and GC-derived autoimmune diseases (95–97), suggesting their involvement in the enhancement of GC-derived human diseases. Other miRNAs that positively regulate GC responses and have been found to promote both autoimmunity and B-cell derived neoplasia include miR-29 and miR-21 (**Table 1**). Further studies are required to establish whether the switched autoantibodies generated in the context of positive GC miRNA regulator overexpression are derived from GC-derived plasma cells or are also generated from extrafollicular plasma cells.

Several miRNAs that negatively regulate the GC reaction have the opposite effect on B cell neoplasia and autoimmunity development, limiting the generation of GC-derived diseases (**Table 1**). miR-28 and miR-146a, well-characterized negative regulators of GC responses (33–35, 52), have both been found to exert tumor suppressor activity in B cell lymphoma development by limiting cell proliferation, promoting cell death and regulating cell signaling (33, 71, 73, 74) (**Table 1**). However, protection against autoimmune diseases has only been explored for miR-146a, which inhibits autoimmunity, anti-dsDNA auto-antibody production (72), and spontaneous GC reactions (52) counterregulating miR-155 targets in Tfh cells (62) and through B cell-intrinsic mechanisms, likely by targeting CD40 signaling pathway components (35). Nevertheless, GC B cell miR-146a expression needs to be tightly regulated because forced overexpression promotes a lymphoproliferative syndrome *via Fas* downregulation (76). Thus, both superabundant or insufficient miR-146a expression are harmful for GC homeostasis.

Overall, these studies show that regulated miRNA expression is required to ensure proper GC responses and that GC-derived dysfunctions caused by miRNA alterations frequently lead to the development of both autoimmunity and B cell neoplasia through the disruption of post-transcriptional control mechanisms required for the maintenance of GC homeostasis, regulated cell signaling, cell death and proliferation. Further studies are needed to characterize with more detail the molecular mechanisms leading to both neoplastic transformation and autoimmunity caused by miRNA-dependent GC gene expression dysregulation.

## Conclusions and Perspective

Studies by many groups in the field have shown that miRNAs play a key role in GC-response regulation and are required to prevent GC-derived autoimmunity and B cell neoplasia. The description of the role of dysregulated miRNAs in mature B cell oncogenic transformation and GC-derived autoimmunity has led to the clinical use of miRNAs as disease biomarkers with prognostic and predictive value and to the identification of targets for miRNA-based therapy (97, 98). The mechanisms leading to dysregulated miRNA expression in GC cells are poorly understood, and their characterization will likely provide new opportunities for therapeutic intervention. Strategies to restore or inhibit dysregulated miRNA expression have already established the therapeutic potential of miRNA modulation in *in vivo* models of GC-derived B cell neoplasia and autoimmunity (33, 44, 56, 68, 99–105). Moreover, synthetic miRNA mimics or anti-miR molecules are suitable for the generation of miRNA-based drugs that can be coupled to different types of nanocarriers and conjugates for effective delivery [reviewed in (106)].

The unique molecular features of miRNAs make them attractive tools for the development of miRNA-based therapies, and miRNA-based drugs are currently being tested in clinical trials for several diseases, including different types of cancer [reviewed in (97)]. This emerging and promising field faces a number of challenges regarding the clinical translation of miRNA-based therapies for B cell neoplasia and autoimmunity. Major challenges include i) the development of cell-type specific miRNA-based drug targeting approaches to improve specificity and reduce toxicity derived from miRNA delivery to healthy cells and ii) the development of models of human mature B cell neoplasia and GC-derived autoimmunity that faithfully recapitulate human disease to improve pre-clinical testing. The rapid pace of research in the field ensures the continuing excitement and expectations in building the path from basic science to translational miRNA-mediated GC regulation.

## Author Contributions

TF, IS and VY contributed substantially to the content and structure of this review. All authors contributed to the article and approved the submitted version.

## Funding

Our work is supported by the Spanish Ministerio de Ciencia e Innovación (PID2019-107551RB-I00). TF is supported by a PhD fellowship from the Spanish Ministerio de Ciencia, Innovacion y Universidades (BES-2017-079759), VY by funding from the Universidad Complutense de Madrid, and IS by a student research grant from the Ministerio de Educación y Formación Profesional.

## Conflict of Interest

A European Patent Application titled ‘miRNA compositions for the treatment of mature B cell neoplasms’ EP16722679, EP17382740 was filed on March 3 2017.

## References

[1] Victora GD Nussenzweig MC. Germinal Centers. Annu Rev Immunol (2012) 30:429–57. 10.1146/annurev-immunol-020711-075032 22224772

[2] Mesin L Ersching J Victora GD. Germinal Center B Cell Dynamics. Immunity (2016) 45:471–82. 10.1016/j.immuni.2016.09.001 PMC5123673 27653600

[3] Vinuesa CG Linterman MA Yu D MacLennan IC. Follicular Helper T Cells. Annu Rev Immunol (2016) 34:335–68. 10.1146/annurev-immunol-041015-055605 26907215

[4] Stebegg M Kumar SD Silva-Cayetano A Fonseca VR Linterman MA Graca L. Regulation of the Germinal Center Response. Front Immunol (2018) 9:2469. 10.3389/fimmu.2018.02469 30410492 PMC6209676

[5] Song S Matthias PD. The Transcriptional Regulation of Germinal Center Formation. Front Immunol (2018) 9:2026. 10.3389/fimmu.2018.02026 30233601 PMC6134015

[6] Gebert LFR MacRae IJ. Regulation of Microrna Function in Animals. Nat Rev Mol Cell Biol (2019) 20:21–37. 10.1038/s41580-018-0045-7 30108335 PMC6546304

[7] Guo WT Wang Y. Dgcr8 Knockout Approaches to Understand Microrna Functions in Vitro and in Vivo. Cell Mol Life Sci (2019) 76:1697–711. 10.1007/s00018-019-03020-9 PMC11105204 30694346

[8] Park CY Choi YS McManus MT. Analysis of Microrna Knockouts in Mice. Hum Mol Genet (2010) 19:R169–75. 10.1093/hmg/ddq367 PMC2981466 20805106

[9] Xu S Guo K Zeng Q Huo J Lam KP. The Rnase III Enzyme Dicer is Essential for Germinal Center B-Cell Formation. Blood (2012) 119:767–76. 10.1182/blood-2011-05-355412 22117047

[10] Baumjohann D Kageyama R Clingan JM Morar MM Patel S de Kouchkovsky D. The Microrna Cluster Mir-17 Approximately 92 Promotes TFH Cell Differentiation and Represses Subset-Inappropriate Gene Expression. Nat Immunol (2013) 14:840–8. 10.1038/ni.2642 PMC3720769 23812098

[11] Fernandez-Messina L Rodriguez-Galan A de Yebenes VG Gutierrez-Vazquez C Tenreiro S Seabra MC. Transfer of Extracellular Vesicle-Microrna Controls Germinal Center Reaction and Antibody Production. EMBO Rep (2020) 21:e48925. 10.15252/embr.201948925 32073750 PMC7132182

[12] Thai TH Calado DP Casola S Ansel KM Xiao C Xue Y. Regulation of the Germinal Center Response by Microrna-155. Science (2007) 316:604–8. 10.1126/science.1141229 17463289

[13] Tam W. Identification and Characterization of Human BIC, a Gene on Chromosome 21 That Encodes a Noncoding RNA. Gene (2001) 274:157–67. 10.1016/S0378-1119(01)00612-6 11675008

[14] Basso K Sumazin P Morozov P Schneider C Maute RL Kitagawa Y. Identification of the Human Mature B Cell Mirnome. Immunity (2009) 30:744–52. 10.1016/j.immuni.2009.03.017 PMC2764486 19446474

[15] Kuchen S Resch W Yamane A Kuo N Li Z Chakraborty T. Regulation of Microrna Expression and Abundance During Lymphopoiesis. Immunity (2010) 32:828–39. 10.1016/j.immuni.2010.05.009 PMC2909788 20605486

[16] Rodriguez A Vigorito E Clare S Warren MV Couttet P Soond DR. Requirement of Bic/Microrna-155 for Normal Immune Function. Science (2007) 316:608–11. 10.1126/science.1139253 PMC2610435 17463290

[17] Vigorito E Perks KL Abreu-Goodger C Bunting S Xiang Z Kohlhaas S. Microrna-155 Regulates the Generation of Immunoglobulin Class-Switched Plasma Cells. Immunity (2007) 27:847–59. 10.1016/j.immuni.2007.10.009 PMC4135426 18055230

[18] Arbore G Henley T Biggins L Andrews S Vigorito E Turner M. Microrna-155 is Essential for the Optimal Proliferation and Survival of Plasmablast B Cells. Life Sci Alliance (2019) 2(3):e201800244. 10.26508/lsa.201800244 31097471 PMC6524163

[19] Nakagawa R Leyland R Meyer-Hermann M Lu D Turner M Arbore G. Microrna-155 Controls Affinity-Based Selection by Protecting C-MYC+ B Cells From Apoptosis. J Clin Invest (2016) 126:377–88. 10.1172/JCI82914 PMC4701536 26657861

[20] Lu D Nakagawa R Lazzaro S Staudacher P Abreu-Goodger C Henley T. The Mir-155-PU.1 Axis Acts on Pax5 to Enable Efficient Terminal B Cell Differentiation. J Exp Med (2014) 211:2183–98. 10.1084/jem.20140338 PMC4203942 25288398

[21] Teng G Hakimpour P Landgraf P Rice A Tuschl T Casellas R. Microrna-155 is a Negative Regulator of Activation-Induced Cytidine Deaminase. Immunity (2008) 28:621–9. 10.1016/j.immuni.2008.03.015 PMC2430982 18450484

[22] Dorsett Y McBride KM Jankovic M Gazumyan A Thai TH Robbiani DF. Microrna-155 Suppresses Activation-Induced Cytidine Deaminase-Mediated Myc-Igh Translocation. Immunity (2008) 28:630–8. 10.1016/j.immuni.2008.04.002 PMC2713656 18455451

[23] Phan RT Saito M Kitagawa Y Means AR Dalla-Favera R. Genotoxic Stress Regulates Expression of the Proto-Oncogene Bcl6 in Germinal Center B Cells. Nat Immunol (2007) 8:1132–9. 10.1038/ni1508 17828269

[24] Phan RT Dalla-Favera R. The BCL6 Proto-Oncogene Suppresses P53 Expression in Germinal-Centre B Cells. Nature (2004) 432:635–9. 10.1038/nature03147 15577913

[25] Basso K Schneider C Shen Q Holmes AB Setty M Leslie C. BCL6 Positively Regulates AID and Germinal Center Gene Expression Via Repression of Mir-155. J Exp Med (2012) 209:2455–65. 10.1084/jem.20121387 PMC3526356 23166356

[26] Basso K Dalla-Favera R. Roles of BCL6 in Normal and Transformed Germinal Center B Cells. Immunol Rev (2012) 247:172–83. 10.1111/j.1600-065X.2012.01112.x 22500840

[27] Bouamar H Jiang D Wang L Lin AP Ortega M Aguiar RC. Microrna 155 Control of P53 Activity is Context Dependent and Mediated by Aicda and Socs1. Mol Cell Biol (2015) 35:1329–40. 10.1128/MCB.01446-14 PMC4372698 25645925

[28] de Yebenes VG Belver L Pisano DG Gonzalez S Villasante A Croce C. Mir-181b Negatively Regulates Activation-Induced Cytidine Deaminase in B Cells. J Exp Med (2008) 205:2199–206. 10.1084/jem.20080579 PMC2556787 18762567

[29] de Yebenes VG Bartolome-Izquierdo N Nogales-Cadenas R Perez-Duran P Mur SM Martinez N. Mir-217 is an Oncogene That Enhances the Germinal Center Reaction. Blood (2014) 124:229–39. 10.1182/blood-2013-12-543611 24850757

[30] Porstner M Winkelmann R Daum P Schmid J Pracht K Corte-Real J. Mir-148a Promotes Plasma Cell Differentiation and Targets the Germinal Center Transcription Factors Mitf and Bach2. Eur J Immunol (2015) 45:1206–15. 10.1002/eji.201444637 25678371

[31] Iqbal J Shen Y Huang X Liu Y Wake L Liu C. Global Microrna Expression Profiling Uncovers Molecular Markers for Classification and Prognosis in Aggressive B-Cell Lymphoma. Blood (2015) 125:1137–45. 10.1182/blood-2014-04-566778 PMC4326773 25498913

[32] Schneider C Setty M Holmes AB Maute RL Leslie CS Mussolin L. Microrna 28 Controls Cell Proliferation and is Down-Regulated in B-Cell Lymphomas. Proc Natl Acad Sci USA (2014) 111:8185–90. 10.1073/pnas.1322466111 PMC4050621 24843176

[33] Bartolome-Izquierdo N de Yebenes VG Alvarez-Prado AF Mur SM Lopez Del Olmo JA Roa S. Mir-28 Regulates the Germinal Center Reaction and Blocks Tumor Growth in Preclinical Models of Non-Hodgkin Lymphoma. Blood (2017) 129:2408–19. 10.1182/blood-2016-08-731166 PMC5437734 28188132

[34] Pratama A Srivastava M Williams NJ Papa I Lee SK Dinh XT. Microrna-146a Regulates ICOS-ICOSL Signalling to Limit Accumulation of T Follicular Helper Cells and Germinal Centres. Nat Commun (2015) 6:6436. 10.1038/ncomms7436 25743066 PMC4366510

[35] Cho S Lee HM Yu IS Choi YS Huang HY Hashemifar SS. Differential Cell-Intrinsic Regulations of Germinal Center B and T Cells by Mir-146a and Mir-146b. Nat Commun (2018) 9:2757. 10.1038/s41467-018-05196-3 30013024 PMC6048122

[36] Malumbres R Sarosiek KA Cubedo E Ruiz JW Jiang X Gascoyne RD. Differentiation Stage-Specific Expression of Micrornas in B Lymphocytes and Diffuse Large B-Cell Lymphomas. Blood (2009) 113:3754–64. 10.1182/blood-2008-10-184077 PMC2670792 19047678

[37] Gururajan M Haga CL Das S Leu CM Hodson D Josson S. Microrna 125b Inhibition of B Cell Differentiation in Germinal Centers. Int Immunol (2010) 22:583–92. 10.1093/intimm/dxq042 PMC2892362 20497960

[38] Tsai DY Hung KH Lin IY Su ST Wu SY Chung CH. Uncovering Microrna Regulatory Hubs That Modulate Plasma Cell Differentiation. Sci Rep (2015) 5:17957. 10.1038/srep17957 26655851 PMC4675970

[39] Morelli E Leone E Cantafio ME Di Martino MT Amodio N Biamonte L. Selective Targeting of IRF4 by Synthetic Microrna-125b-5p Mimics Induces Anti-Multiple Myeloma Activity in Vitro and in Vivo. Leukemia (2015) 29:2173–83. 10.1038/leu.2015.124 PMC4635336 25987254

[40] Nie K Gomez M Landgraf P Garcia JF Liu Y Tan LH. Microrna-Mediated Down-Regulation of PRDM1/Blimp-1 in Hodgkin/Reed-Sternberg Cells: A Potential Pathogenetic Lesion in HODGKIN Lymphomas. Am J Pathol (2008) 173:242–52. 10.2353/ajpath.2008.080009 PMC2438301 18583325

[41] Nie K Zhang T Allawi H Gomez M Liu Y Chadburn A. Epigenetic Down-Regulation of the Tumor Suppressor Gene PRDM1/Blimp-1 in Diffuse Large B Cell Lymphomas: A Potential Role of the Microrna Let-7. Am J Pathol (2010) 177:1470–9. 10.2353/ajpath.2010.091291 PMC2928978 20651244

[42] Zhang J Jima DD Jacobs C Fischer R Gottwein E Huang G. Patterns of Microrna Expression Characterize Stages of Human B-Cell Differentiation. Blood (2009) 113:4586–94. 10.1182/blood-2008-09-178186 PMC2680365 19202128

[43] Huang X Zhou X Wang Z Li F Liu F Zhong L. CD99 Triggers Upregulation of Mir-9-Modulated PRDM1/BLIMP1 in Hodgkin/Reed-Sternberg Cells and Induces Redifferentiation. Int J Cancer (2012) 131:E382–94. 10.1002/ijc.26503 22020966

[44] White CA Pone EJ Lam T Tat C Hayama KL Li G. Histone Deacetylase Inhibitors Upregulate B Cell Micrornas That Silence AID and Blimp-1 Expression for Epigenetic Modulation of Antibody and Autoantibody Responses. J Immunol (2014) 193:5933–50. 10.4049/jimmunol.1401702 PMC4258531 25392531

[45] Shen T Sanchez HN Zan H Casali P. Genome-Wide Analysis Reveals Selective Modulation of Micrornas and Mrnas by Histone Deacetylase Inhibitor in B Cells Induced to Undergo Class-Switch DNA Recombination and Plasma Cell Differentiation. Front Immunol (2015) 6:627. 10.3389/fimmu.2015.00627 26697020 PMC4677488

[46] Moroney JB Chupp DP Xu Z Zan H Casali P. Epigenetics of the Antibody and Autoantibody Response. Curr Opin Immunol (2020) 67:75–86. 10.1016/j.coi.2020.09.004 33176228 PMC7744442

[47] Moroney JB Vasudev A Pertsemlidis A Zan H Casali P. Integrative Transcriptome and Chromatin Landscape Analysis Reveals Distinct Epigenetic Regulations in Human Memory B Cells. Nat Commun (2020) 11:5435. 10.1038/s41467-020-19242-6 33116135 PMC7595102

[48] Kang SG Liu WH Lu P Jin HY Lim HW Shepherd J. Micrornas of the Mir-17 Approximately 92 Family are Critical Regulators of T(FH) Differentiation. Nat Immunol (2013) 14:849–57. 10.1038/ni.2648 PMC3740954 23812097

[49] Wu T Wieland A Lee J Hale JS Han JH Xu X. Cutting Edge: Mir-17-92 is Required for Both CD4 Th1 and T Follicular Helper Cell Responses During Viral Infection. J Immunol (2015) 195:2515–9. 10.4049/jimmunol.1500317 PMC5053620 26276869

[50] Jin HY Oda H Lai M Skalsky RL Bethel K Shepherd J. Microrna-17~92 Plays a Causative Role in Lymphomagenesis by Coordinating Multiple Oncogenic Pathways. EMBO J (2013) 32:2377–91. 10.1038/emboj.2013.178 PMC3771343 23921550

[51] Xiao C Srinivasan L Calado DP Patterson HC Zhang B Wang J. Lymphoproliferative Disease and Autoimmunity in Mice With Increased Mir-17-92 Expression in Lymphocytes. Nat Immunol (2008) 9:405–14. 10.1038/ni1575 PMC2533767 18327259

[52] Hu R Kagele DA Huffaker TB Runtsch MC Alexander M Liu J. Mir-155 Promotes T Follicular Helper Cell Accumulation During Chronic, Low-Grade Inflammation. Immunity (2014) 41:605–19. 10.1016/j.immuni.2014.09.015 PMC4657560 25367574

[53] Costinean S Zanesi N Pekarsky Y Tili E Volinia S Heerema N. Pre-B Cell Proliferation and Lymphoblastic Leukemia/High-Grade Lymphoma in E(Mu)-Mir155 Transgenic Mice. Proc Natl Acad Sci USA (2006) 103:7024–9. 10.1073/pnas.0602266103 PMC1459012 16641092

[54] Costinean S Sandhu SK Pedersen IM Tili E Trotta R Perrotti D. Src Homology 2 Domain-Containing Inositol-5-Phosphatase and CCAAT Enhancer-Binding Protein Beta are Targeted by Mir-155 in B Cells of Emicro-Mir-155 Transgenic Mice. Blood (2009) 114:1374–82. 10.1182/blood-2009-05-220814 PMC2727407 19520806

[55] Pedersen IM Otero D Kao E Miletic AV Hother C Ralfkiaer E. Onco-Mir-155 Targets SHIP1 to Promote Tnfalpha-Dependent Growth of B Cell Lymphomas. EMBO Mol Med (2009) 1:288–95. 10.1002/emmm.200900028 PMC2771872 19890474

[56] Babar IA Cheng CJ Booth CJ Liang X Weidhaas JB Saltzman WM. Nanoparticle-Based Therapy in an in Vivo Microrna-155 (Mir-155)-Dependent Mouse Model of Lymphoma. Proc Natl Acad Sci USA (2012) 109:E1695–704. 10.1073/pnas.1201516109 PMC3387084 22685206

[57] Kurowska-Stolarska M Alivernini S Ballantine LE Asquith DL Millar NL Gilchrist DS. Microrna-155 as a Proinflammatory Regulator in Clinical and Experimental Arthritis. Proc Natl Acad Sci USA (2011) 108:11193–8. 10.1073/pnas.1019536108 PMC3131377 21690378

[58] Thai TH Patterson HC Pham DH Kis-Toth K Kaminski DA Tsokos GC. Deletion of Microrna-155 Reduces Autoantibody Responses and Alleviates Lupus-Like Disease in the Fas(Lpr) Mouse. Proc Natl Acad Sci USA (2013) 110:20194–9. 10.1073/pnas.1317632110 PMC3864325 24282294

[59] Xin Q Li J Dang J Bian X Shan S Yuan J. Mir-155 Deficiency Ameliorates Autoimmune Inflammation of Systemic Lupus Erythematosus by Targeting S1pr1 in Faslpr/Lpr Mice. J Immunol (2015) 194:5437–45. 10.4049/jimmunol.1403028 25911753

[60] Alivernini S Kurowska-Stolarska M Tolusso B Benvenuto R Elmesmari A Canestri S. Microrna-155 Influences B-Cell Function Through PU.1 in Rheumatoid Arthritis. Nat Commun (2016) 7:12970. 10.1038/ncomms12970 27671860 PMC5052655

[61] Liu WH Kang SG Huang Z Wu CJ Jin HY Maine CJ. A Mir-155-Peli1-C-Rel Pathway Controls the Generation and Function of T Follicular Helper Cells. J Exp Med (2016) 213:1901–19. 10.1084/jem.20160204 PMC4995083 27481129

[62] Chen J Tian J Tang X Rui K Ma J Mao C. Mir-346 Regulates CD4(+)CXCR5(+) T Cells in the Pathogenesis of Graves’ Disease. Endocrine (2015) 49:752–60. 10.1007/s12020-015-0546-5 25666935

[63] van Nieuwenhuijze A Dooley J Humblet-Baron S Sreenivasan J Koenders M Schlenner SM. Defective Germinal Center B-Cell Response and Reduced Arthritic Pathology in Microrna-29a-Deficient Mice. Cell Mol Life Sci (2017) 74:2095–106. 10.1007/s00018-017-2456-6 PMC11107729 28124096

[64] Santanam U Zanesi N Efanov A Costinean S Palamarchuk A Hagan JP. Chronic Lymphocytic Leukemia Modeled in Mouse by Targeted Mir-29 Expression. Proc Natl Acad Sci USA (2010) 107:12210–5. 10.1073/pnas.1007186107 PMC2901490 20566844

[65] Yan Y Deng X Ning X Li F Cao J. Pathogenic Mechanism of Mir-21 in Autoimmune Lymphoid Hyperplasia Syndrome. Oncol Lett (2017) 13:4734–40. 10.3892/ol.2017.6039 PMC5452897 28588726

[66] Barnes NA Stephenson S Cocco M Tooze RM Doody GM. BLIMP-1 and STAT3 Counterregulate Microrna-21 During Plasma Cell Differentiation. J Immunol (2012) 189:253–60. 10.4049/jimmunol.1101563 22634616

[67] Medina PP Nolde M Slack FJ. Oncomir Addiction in an in Vivo Model of Microrna-21-Induced Pre-B-Cell Lymphoma. Nature (2010) 467:86–90. 10.1038/nature09284 20693987

[68] Garchow BG Bartulos Encinas O Leung YT Tsao PY Eisenberg RA Caricchio R. Silencing of Microrna-21 in Vivo Ameliorates Autoimmune Splenomegaly in Lupus Mice. EMBO Mol Med (2011) 3:605–15. 10.1002/emmm.201100171 PMC3258486 21882343

[69] Gu L Song G Chen L Nie Z He B Pan Y. Inhibition of Mir-21 Induces Biological and Behavioral Alterations in Diffuse Large B-Cell Lymphoma. Acta Haematol (2013) 130:87–94. 10.1159/000346441 23548551

[70] Go H Jang JY Kim PJ Kim YG Nam SJ Paik JH. Microrna-21 Plays an Oncogenic Role by Targeting FOXO1 and Activating the PI3K/AKT Pathway in Diffuse Large B-Cell Lymphoma. Oncotarget (2015) 6:15035–49. 10.18632/oncotarget.3729 PMC4558134 25909227

[71] Zhao JL Rao DS Boldin MP Taganov KD O’Connell RM Baltimore D. NF-Kappab Dysregulation in Microrna-146a-Deficient Mice Drives the Development of Myeloid Malignancies. Proc Natl Acad Sci USA (2011) 108:9184–9. 10.1073/pnas.1105398108 PMC3107319 21576471

[72] Boldin MP Taganov KD Rao DS Yang L Zhao JL Kalwani M. Mir-146a is a Significant Brake on Autoimmunity, Myeloproliferation, and Cancer in Mice. J Exp Med (2011) 208:1189–201. 10.1084/jem.20101823 PMC3173243 21555486

[73] Mitsumura T Ito Y Chiba T Matsushima T Kurimoto R Tanaka Y. Ablation of Mir-146b in Mice Causes Hematopoietic Malignancy. Blood Adv (2018) 2:3483–91. 10.1182/bloodadvances.2018017954 PMC6290096 30530754

[74] Contreras JR Palanichamy JK Tran TM Fernando TR Rodriguez-Malave NI Goswami N. Microrna-146a Modulates B-Cell Oncogenesis by Regulating Egr1. Oncotarget (2015) 6:11023–37. 10.18632/oncotarget.3433 PMC4484436 25906746

[75] Wang H Li X Li T Wang L Wu X Liu J. Multiple Roles of Microrna-146a in Immune Responses and Hepatocellular Carcinoma. Oncol Lett (2019) 18:5033–42. 10.3892/ol.2019.10862 PMC6781720 31612014

[76] Guo Q Zhang J Li J Zou L Zhang J Xie Z. Forced Mir-146a Expression Causes Autoimmune Lymphoproliferative Syndrome in Mice Via Downregulation of Fas in Germinal Center B Cells. Blood (2013) 121:4875–83. 10.1182/blood-2012-08-452425 23645835

[77] Xu H Li X Liu D Li J Zhang X Chen X. Follicular T-Helper Cell Recruitment Governed by Bystander B Cells and ICOS-Driven Motility. Nature (2013) 496:523–7. 10.1038/nature12058 23619696

[78] Yu D Tan AH Hu X Athanasopoulos V Simpson N Silva DG. Roquin Represses Autoimmunity by Limiting Inducible T-Cell Co-Stimulator Messenger RNA. Nature (2007) 450:299–303. 10.1038/nature06253 18172933

[79] Athanasopoulos V Barker A Yu D Tan AH Srivastava M Contreras N. The ROQUIN Family of Proteins Localizes to Stress Granules Via the ROQ Domain and Binds Target Mrnas. FEBS J (2010) 277:2109–27. 10.1111/j.1742-4658.2010.07628.x 20412057

[80] Gigoux M Shang J Pak Y Xu M Choe J Mak TW. Inducible Costimulator Promotes Helper T-Cell Differentiation Through Phosphoinositide 3-Kinase. Proc Natl Acad Sci USA (2009) 106:20371–6. 10.1073/pnas.0911573106 PMC2787139 19915142

[81] Rolf J Bell SE Kovesdi D Janas ML Soond DR Webb LM. Phosphoinositide 3-Kinase Activity in T Cells Regulates the Magnitude of the Germinal Center Reaction. J Immunol (2010) 185:4042–52. 10.4049/jimmunol.1001730 20826752

[82] Yu D Rao S Tsai LM Lee SK He Y Sutcliffe EL. The Transcriptional Repressor Bcl-6 Directs T Follicular Helper Cell Lineage Commitment. Immunity (2009) 31:457–68. 10.1016/j.immuni.2009.07.002 19631565

[83] Essig K Hu D Guimaraes JC Alterauge D Edelmann S Raj T. Roquin Suppresses the PI3K-Mtor Signaling Pathway to Inhibit T Helper Cell Differentiation and Conversion of Treg to Tfr Cells. Immunity (2017) 47:1067–82.e12. 10.1016/j.immuni.2017.11.008 29246441

[84] Ripamonti A Provasi E Lorenzo M De Simone M Ranzani V Vangelisti S. Repression of Mir-31 by BCL6 Stabilizes the Helper Function of Human Follicular Helper T Cells. Proc Natl Acad Sci USA (2017) 114:12797–802. 10.1073/pnas.1705364114 PMC5715737 29133396

[85] Deenick EK Ma CS. The Regulation and Role of T Follicular Helper Cells in Immunity. Immunology (2011) 134:361–7. 10.1111/j.1365-2567.2011.03487.x PMC3230790 22043829

[86] Cannons JL Qi H Lu KT Dutta M Gomez-Rodriguez J Cheng J. Optimal Germinal Center Responses Require a Multistage T Cell:B Cell Adhesion Process Involving Integrins, SLAM-Associated Protein, and CD84. Immunity (2010) 32:253–65. 10.1016/j.immuni.2010.01.010 PMC2830297 20153220

[87] Kashiwakuma D Suto A Hiramatsu Y Ikeda K Takatori H Suzuki K. B and T Lymphocyte Attenuator Suppresses IL-21 Production From Follicular Th Cells and Subsequent Humoral Immune Responses. J Immunol (2010) 185:2730–6. 10.4049/jimmunol.0903839 20660710

[88] Takahashi H Kanno T Nakayamada S Hirahara K Sciume G Muljo SA. TGF-Beta and Retinoic Acid Induce the Microrna Mir-10a, Which Targets Bcl-6 and Constrains the Plasticity of Helper T Cells. Nat Immunol (2012) 13:587–95. 10.1038/ni.2286 PMC3499969 22544395

[89] Quezada SA Jarvinen LZ Lind EF Noelle RJ. CD40/CD154 Interactions At the Interface of Tolerance and Immunity. Annu Rev Immunol (2004) 22:307–28. 10.1146/annurev.immunol.22.012703.104533 15032580

[90] Perez-Melgosa M Hollenbaugh D Wilson CB. Cutting Edge: CD40 Ligand is a Limiting Factor in the Humoral Response to T Cell-Dependent Antigens. J Immunol (1999) 163:1123–7. 10415005

[91] Vinuesa CG Sanz I Cook MC. Dysregulation of Germinal Centres in Autoimmune Disease. Nat Rev Immunol (2009) 9:845–57. 10.1038/nri2637 19935804

[92] Elsner RA Shlomchik MJ. Germinal Center and Extrafollicular B Cell Responses in Vaccination, Immunity, and Autoimmunity. Immunity (2020) 53:1136–50. 10.1016/j.immuni.2020.11.006 PMC7748291 33326765

[93] Mlynarczyk C Fontan L Melnick A. Germinal Center-Derived Lymphomas: The Darkest Side of Humoral Immunity. Immunol Rev (2019) 288:214–39. 10.1111/imr.12755 PMC6518944 30874354

[94] Wen Z Xu L Chen X Xu W Yin Z Gao X. Autoantibody Induction by DNA-Containing Immune Complexes Requires HMGB1 With the TLR2/Microrna-155 Pathway. J Immunol (2013) 190:5411–22. 10.4049/jimmunol.1203301 23616573

[95] Kuo G Wu CY Yang HY. Mir-17-92 Cluster and Immunity. J Formos Med Assoc (2019) 118:2–6. 10.1016/j.jfma.2018.04.013 29857952

[96] Leng RX Pan HF Qin WZ Chen GM Ye DQ. Role of Microrna-155 in Autoimmunity. Cytokine Growth Factor Rev (2011) 22:141–7. 10.1016/j.cytogfr.2011.05.002 21703910

[97] Fuertes T Ramiro AR de Yebenes VG. Mirna-Based Therapies in B Cell Non-Hodgkin Lymphoma. Trends Immunol (2020) 41:932–47. 10.1016/j.it.2020.08.006 32888820

[98] Long H Wang X Chen Y Wang L Zhao M Lu Q. Dysregulation of Micrornas in Autoimmune Diseases: Pathogenesis, Biomarkers and Potential Therapeutic Targets. Cancer Lett (2018) 428:90–103. 10.1016/j.canlet.2018.04.016 29680223

[99] Zhang Y Roccaro AM Rombaoa C Flores L Obad S Fernandes SM. LNA-Mediated Anti-Mir-155 Silencing in Low-Grade B-Cell Lymphomas. Blood (2012) 120:1678–86. 10.1182/blood-2012-02-410647 22797699

[100] Zhu FQ Zeng L Tang N Tang YP Zhou BP Li FF. Microrna-155 Downregulation Promotes Cell Cycle Arrest and Apoptosis in Diffuse Large B-Cell Lymphoma. Oncol Res (2016) 24:415–27. 10.3727/096504016X14685034103473 PMC7838747 28281962

[101] Cheng CJ Bahal R Babar IA Pincus Z Barrera F Liu C. Microrna Silencing for Cancer Therapy Targeted to the Tumour Microenvironment. Nature (2015) 518:107–10. 10.1038/nature13905 PMC4367962 25409146

[102] Leone E Morelli E Di Martino MT Amodio N Foresta U Gulla A. Targeting Mir-21 Inhibits in Vitro and in Vivo Multiple Myeloma Cell Growth. Clin Cancer Res (2013) 19:2096–106. 10.1158/1078-0432.CCR-12-3325 PMC4147955 23446999

[103] Su YL Wang X Mann M Adamus TP Wang D Moreira DF. Myeloid Cell-Targeted Mir-146a Mimic Inhibits NF-Kappab-Driven Inflammation and Leukemia Progression in Vivo. Blood (2020) 135:167–80. 10.1182/blood.2019002045 PMC6966933 31805184

[104] Zhang J Jia G Liu Q Hu J Yan M Yang B. Silencing Mir-146a Influences B Cells and Ameliorates Experimental Autoimmune Myasthenia Gravis. Immunology (2015) 144:56–67. 10.1111/imm.12347 24962817 PMC4264910

[105] Casali P Shen T Xu Y Qiu Z Chupp DP Im J. Estrogen Reverses HDAC Inhibitor-Mediated Repression of Aicda and Class-Switching in Antibody and Autoantibody Responses by Downregulation of Mir-26a. Front Immunol (2020) 11:491. 10.3389/fimmu.2020.00491 32265934 PMC7105609

[106] Rupaimoole R Slack FJ. Microrna Therapeutics: Towards a New Era for the Management of Cancer and Other Diseases. Nat Rev Drug Discovery (2017) 16:203–22. 10.1038/nrd.2016.246 28209991

